# The Ultrastructural Identity of Alzheimer's Pathology: Lessons from Animal Models

**DOI:** 10.4172/2329-6895.1000e111

**Published:** 2014-04-28

**Authors:** Krikor Dikranian, David M. Holtzman

**Affiliations:** 1 Department of Anatomy and Neurobiology, Hope Center for Neurological Disorders, Washington University School of Medicine in Saint Louis, USA; 2 Department of Neurology, Hope Center for Neurological Disorders, Knight Alzheimer's Disease Research Center, Washington University School of Medicine in Saint Louis, USA

## Editorial

Dr. Alois Alzheimer was an accomplished histopathologist. At the time of his November 1906 lecture in Tübingen, Germany, where he presented the case of his patient Augustine D, he had spent many years in the histology lab. Four years earlier, the “Bielschowsky” silver staining method was developed and nearly a decade before Santiago Ramón y Cajal had perfected Camillo Golgi's silver staining method. These techniques were crucial for the identification of the neuritic plaques and tangles in Frau Augustine's brain. Almost 60 years later, after thousands of histopathological slides were examined by conventional light microscopy, electron microscopy (EM) began to reveal new facets of this disease by unraveling the submicroscopic structure of AD pathology. Terry, Kidd and Wisniewski published the first detailed reports of postmortem AD brains ultrastructure. Senile plaques were described of being composed of extracellular deposits of an amyloid-β (Aβ) “central fibrillar core”, surrounded by “axons and dendrites filled with an excess of neurofibrils”, and “cell processes filled with dense bodies” along with a halo of glial cells. The earliest signs of plaque formation, the “diffuse plaque” and amyloid deposition in AD-related angiopathy were later described. The progressive decrease in synaptic numbers in humans was documented. Neurofibrillary tangles (NFT) were shown to be formed by paired helical filaments (PHF). Scanning EM was also applied with great success for the study of PHF. After the identification of plaque filaments it took two decades before their major components were known. Since 1996, only few systemic human EM studies have been published.

**Figure 1:**
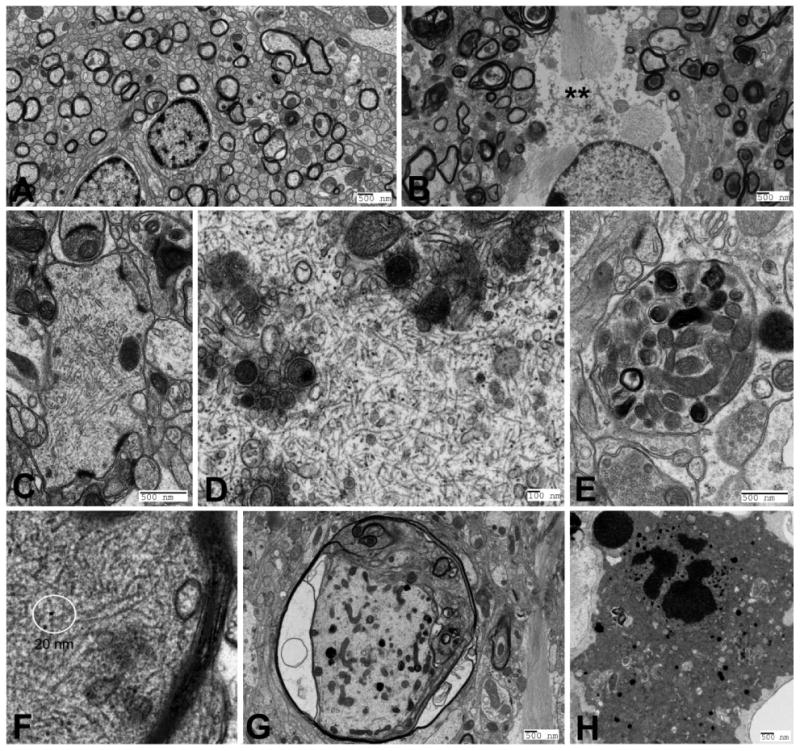
Electron micrographs of 9 month old control (A) non-transgenic and P301S +/- single transgenic mouse (B-H). A and B show the structure of the neuropil in the amygdala. Note numerous degenerating myelinated axons and an activated astroglial cell (**) in B. Images in C, D, and E show accumulation of 20 nm fillaments in a dendritic spine (C), neuronal cell cytoplasm (D) and myelinated axon (F). Images E and G show degenerating unmyelinated (E) and myelinated (G) axons with compaction of organelles and accumulation of fillaments. H. EM image of an apoptotic neuron.

The generation of transgenic (tg) animals as models overexpressing mutant human amyloid precursor protein (APP) and/or presenilin-1 and -2 (PS1, PS2), tg mice expressing human ApoE isoforms, as well as tau, have made substantial contributions to the understanding of AD-type brain pathology and stimulated renewed interest in EM. However, only a few studies have compared tg animal models and AD brains at the ultrastrucural level. The first tg mouse models were based on the overexpression of single or multiple mutant molecules associated with familial AD. In 1996 Masliah and his group compared the pathology found in the PDAPP (platelet-derived growth factor-B promoter driven hAPP minigene) tg mouse line and in AD brains. They concluded that overproduction of human APP with a familial AD mutation is sufficient to cause AD-related degenerative changes and amyloid deposition in single tg mice between 8 and 12 months of age. Mice overexpressing the mutant PS1 alone have not been shown to accumulate Aβ in the brain while co-expression of mutant PS1 with APP has been associated with amyloid deposition. In 2001 Kurt and his group provided a detailed ultrastructural study of these APP/PS1 mice. Plaques were observed in the neuropil and white matter, the amyloid core was surrounded by microglial and astroglial cell processes. Neuronal degeneration of the non-apoptotic type was registered close to plaques. A special type of degenerative feature, the accumulation of autophagic vacuoles was recognized. Plaques were described also in the spinal cord accompanied by massive axonal degeneration in white matter tracts structurally resembling Wallerian type. The ε4 allele of apolipoprotein E (ApoE) is the strongest genetic risk factor for the more common sporadic forms of AD. Our detailed ultrastructural study in APP/PS1 tg mice expressing each one of the three human apolipoprotein isoforms (ApoE2, ApoE3 or ApoE4) revealed mature neuritic plaques in the white and grey matter and robust axonal and synaptic pathology amazingly similar to APP/PS1 tg animals. Interestingly, some of the axoplasmic dystrophic changes were similar to the degenerative changes seen in traumatic brain injury and indicated axonal transport disruption. A hallmark of AD is the intraneuronal accumulation of PHF. Tau protein, a multifunctional microtubule-associated protein, in its aberrant form, aggregates into PHF and loses its microtubule stabilizing function. Tauopathies are a heterogeneous group of dementias sharing a common mechanism, aberrant tau metabolism. The most prevalent tauopathy is AD. Early EM studies in single tau tg mice have shown that overexpression of human four-repeat tau resulted in axonal dystrophy, axoplasmic filament and tau-immunopositive “spheroid” accumulation. Results from 6 month old P301S tau tg mice showed abundance of tau-immunoreacive twisted ribbons and filaments resembling PHF. Early hippocampal synapse loss, prominent astrogliosis and microglial activation was reported as well. EM revealed tangle-like 12-20 nm tau-immonorecative filament accumulation in neurons and their processes, accompanied by neuronal and axonal degeneration. Our own studies in P301S heterozygous tg mice have also shown robust axonal degeneration identical to that seen in APP, APP/PS1 and APP/PS1/ApoE animals. These changes were accompanied by neuronal, axoplasmic and dendritic accumulation of 18-20 nm filaments, apoptotic and non-apoptotic cell degeneration and astroglial activation (Figure 1). Similar results have been reported by others. Hippocampal neurons with abundant 15 nm filaments with wavy and straight characteristics were reported in aged P301L tg mice. EM data of microglial activation and apoptotic glial cell death was observed. In a mouse line bearing two pathogenic tau mutations (P301S and G272V) age-dependent brain atrophy, intraneuronal straight and twisted filament accumulation, axonal dystrophy in the form of organelle compaction, lamellar body accumulation and myelin atrophy was also detected. Interestingly glial cell tau filaments have been described as well. Injection of Aβ42 fibrils into 6-moonths old P301L tg mouse brains produced the characteristic twisted filaments suggesting that Aβ42 fibrils can accelerate NFT formation in vivo. Double transgenic APP and human tau tg mice have shown cognitive impairment, robust NFT formation, neuronal, synapse and memory loss. These results have suggested that Aβ oligomers can induce NFT in the absence of amyloid plaques and the presence of human tau is critical for NFT formation. In 2003 a triple transgenic APP/PS1/tau(P301L) line was introduced. Triple tg mice exhibited synaptic deficits prior to manifestation of pathology and progressively developed amyloid plaques and tangles. Phosphorylated tau-imminopositive processes close to plaques, axonal accumulation of 10-12 nm filaments, and 26 nm tubular structures were observed as well. In a similar transgenic model PHF-1- immunopositive NFT were reported. As expected, EM data were strikingly similar to APP, APP/PS1, APP/PS1/ApoE tg animals.

While no mouse model fully recapitulated the entire neuropathological spectrum of AD, at the EM level tg animal models have replicated all “hallmark” features of Alzheimer's pathology. Existing and new tg models will undoubtedly drive research for understanding mechanisms of amyloid secretion, PHF assembly, the nature of cell degeneration, synapse loss and the enigmatic role of glial involvement. While this quest is on, EM must remain an important “gold standard” like in the early days of modern AD research.

